# Camrelizumab Plus Sorafenib *Versus* Sorafenib Monotherapy for Advanced Hepatocellular Carcinoma: A Retrospective Analysis

**DOI:** 10.3389/fonc.2021.694409

**Published:** 2021-10-19

**Authors:** Qinqin Liu, Nan You, Jing Li, Ke Wu, Xuehui Peng, Zheng Wang, Liang Wang, Yinan Zhu, Lu Zheng

**Affiliations:** ^1^ Department of Hepatobiliary Surgery, The Second Affiliated Hospital of Army Medical University, Chongqing, China; ^2^ Department of Biliary-Pancreatic Surgery, Sun Yat-Sen Memorial Hospital, Sun Yat-Sen University, Guangzhou, China

**Keywords:** hepatocellular carcinoma, camrelizumab, sorafenib, efficacy, safety

## Abstract

**Background:**

Hepatocellular carcinoma (HCC) is a highly aggressive malignancy with poor prognosis. Immunotherapy has gained great interest for various solid tumors due to its promising clinical efficacy. Targeted therapy also plays a crucial role in anticancer treatment. However, studies on the combination of immunotherapy and targeted therapy for advanced HCC are limited. Thus, the objective of this study was to investigate the efficacy and safety of camrelizumab combined with sorafenib in the treatment of advanced HCC.

**Methods:**

From January 2019 to January 2021, 100 consecutive patients with advanced HCC in our hospital were enrolled for this study. Patients were assigned into two groups: a combined-therapy group (camrelizumab + sorafenib) and a sorafenib-only group. Progression-free survival (PFS), overall survival (OS), treatment response, and relevant adverse effects (AEs) were evaluated and recorded.

**Results:**

Of a total of 100 patients, 35 received a combination of camrelizumab and sorafenib, and 65 were treated with sorafenib alone. After 1:1 propensity score matching (PSM), each group had 34 patients. The overall response rate (ORR) of the combined-therapy group was statistically significantly higher than that of the sorafenib-only group (before PSM, *p* = 0.037; after PSM, *p* = 0.010). However, there was no significant difference in disease control rate (DCR) between the two groups (before PSM, *p* = 0.695; after PSM, *p* = 1.000). Patients who received the combination therapy had significantly longer PFS than those who received the sorafenib monotherapy (before PSM, *p* = 0.041; after PSM, *p* = 0.043). However, the two groups exhibited comparable median OS (before PSM, *p* = 0.135; after PSM, *p* = 0.105). Although the combined-therapy group showed a higher incidence of AEs such as thrombocytopenia than the sorafenib-only group after PSM, most of these AEs were easily controlled after treatment.

**Conclusion:**

Camrelizumab plus sorafenib showed favorable efficacy and manageable toxicity for patients with advanced HCC. However, more prospective randomized trials are necessary to further verify the potential clinical benefits of this combination therapy.

## Introduction

Hepatocellular carcinoma (HCC), one of the most lethal malignancies globally, is the fourth-ranked cause of cancer mortality worldwide (1). HCC is often detected at a late stage due to the insidious and asymptomatic progression, which is not amenable to curative interventions such as liver resection, liver transplantation, or radiofrequency ablation (2, 3). Despite tremendous progress has been made in HCC diagnosis and treatment, clinical outcomes of advanced HCC remain disappointing with current available therapeutic modalities (4). Therefore, it is of paramount importance to investigate more effective treatment strategies for patients with advanced HCC.

Multitarget tyrosine kinase inhibitors (TKIs) play a vital role in the clinical management of patients with various types of solid tumors (5). TKIs can suppress tumor proliferation and angiogenesis by targeting vascular endothelial growth factor receptors (VEGFRs), platelet-derived growth factor receptors (PDGFRs), fibroblast growth factor receptors (FGFRs), stem cell factor receptor (KIT), and glial cell-derived neurotrophic factor receptor (RET) (6–8). Of these, sorafenib is the first-line targeted agent for advanced HCC patients that can lead to a median overall survival (OS) of 6.5–10.7 months and a median time to progression of 2.8–5.5 months (9, 10). However, sorafenib is associated with a low response rate of 30% and high risks of acquired drug resistance and disease progression, thus limiting its long-term clinical benefits (11). Although other molecular targeted drugs have been developed, the efficacy of targeted therapy in advanced HCC remains a serious concern.

Immune checkpoint inhibitors (ICIs), which target cytotoxic T-lymphocyte antigen-4 (CTLA-4), programmed cell death protein 1 (PD-1), and programmed cell death protein ligand 1 (PD-L1), show a promising prospect in cancer therapeutics (12). The anti-PD-1 antibodies nivolumab and pembrolizumab have obtained the U.S. Food and Drug Administration (FDA) approval as second-line agents for treating advanced HCC (13, 14). Despite the positive role of ICIs in the treatment of HCC, subsequent phase 3 CheckMate459 (15) and KEYNOTE-240 (16) trials demonstrated that nivolumab (versus placebo) and pembrolizumab (versus sorafenib) failed to show significant survival superiority, indicating the necessity to explore more appropriate systemic treatment strategies to enhance the efficacy of immunotherapy. Angiogenesis inhibitors can impair immunosuppression in the tumor microenvironment and facilitate antitumor efficacy of immune checkpoint inhibitors by affecting T-cell activation, thus providing a strong rationale for combination trials (17). Recently, the combination of atezolizumab and bevacizumab as first-line therapy for unresectable HCC has been approved by the U.S. FDA (18), encouraging further investigation for other potential promising combination treatments.

Hence, the objective of the present study was to evaluate the safety and therapeutic efficacy of a combination therapy using camrelizumab and sorafenib for patients with advanced HCC compared with sorafenib monotherapy.

## Methods

### Patients

A total of 100 patients with advanced HCC who received sorafenib treatment from January 2019 to January 2021 in the Second Affiliated Hospital of Army Medical University were included in this study. The first-line treatment included surgery, ablation, and transarterial chemoembolization (TACE). Inclusion criteria were as follows: (1) male or female patients aged over 18 years; (2) HCC diagnosis was based on histological examination or the criteria of the American Association for the Study of Liver Diseases (AASLD) guidelines (2); (3) those who had Child‐Pugh liver function class A or B; (4) those who had Eastern Cooperative Oncology Group (ECOG) performance status score of 0 or 1; (5) those with the presence of unresectable or metastatic lesions; (6) those with acceptable heart, hepatic, renal, and hematologic functions; (7) those who had estimated life expectancy ≥12 weeks; and (8) at least one measurable target lesion based on the modified Response Evaluation Criteria in Solid Tumors (mRECIST) (19). Exclusion criteria were (1) those who had a previous history of treatment with sorafenib or any other PD-L1/PD-1 antagonists; (2) those with other malignant tumors; (3) those who were pregnant or breastfeeding; and (4) those who had incomplete follow-up data. All patients received routine biochemical tests and radiological examinations preoperatively. This study was approved by the Ethics Committee of The Second Affiliated Hospital of Army Medical University. Written consent was obtained from each patient for the collection of clinical data for research purposes.

### Treatment Protocol

For patients in the sorafenib-only group, sorafenib was administered 400 mg orally twice per day. For those in the combined-therapy group, camrelizumab 200 mg was intravenously administered every 2 weeks and sorafenib 400 mg was given orally once per day. When patients experienced grade 3/4 treatment-related adverse events (AEs), the dose of sorafenib was reduced by 50% or discontinued until the severity of AEs decreased to grade ≤2. Patients were treated until death, disease progression, unacceptable toxicity, or consent withdrawn from this study.

### Endpoints and Assessments

Demographic and clinical data including age, gender, hepatitis B virus (HBV) carrier, liver cirrhosis, ECOG performance score, Child-Pugh score, Barcelona Clinic Liver Cancer (BCLC) stage, alpha-fetoprotein (AFP), total bilirubin (TBIL), albumin (ALB), aspartate aminotransferase (AST), alanine aminotransferase (ALT), platelet count (PLT), white blood cell (WBC), prothrombin time (PT), tumor size, tumor number, macrovascular invasion, extrahepatic metastasis, and previous local regional therapy were recorded. Each patient received CT or MRI evaluation at baseline and every two cycles of treatment (8 weeks) thereafter. Tumor responses were assessed according to the mRECIST and categorized as complete response (CR), partial response (PR), stable disease (SD), or progressive disease (PD).

The primary study endpoint was progression-free survival (PFS). Secondary study endpoints were over survival (OS), objective response rate (ORR), disease control rate (DCR), and AEs. PFS was calculated as the time from the treatment initiation to the date of disease progression or death from any cause. OS was estimated from the treatment initiation to the date of death from any cause or the last follow-up. DCR was calculated as the percentage of patients with CR, PR, or SD. ORR was calculated as the percentage of patients with CR or PR. AEs were assessed and graded according to the Common Terminology Criteria for Adverse Events (CTCAE, version 4.0).

### Statistical Analysis

Propensity score matching (PSM) was conducted for the following variables: age, sex, HBV carrier, liver cirrhosis, ECOG performance score, Child-Pugh stage, BCLC stage, AFP, tumor size, tumor number, macrovascular invasion, extrahepatic metastasis, and previous local regional therapy. Continuous data are expressed as mean ± standard deviation or median with interquartile range and compared using Student’s *t*-test or Mann-Whitney-Wilcoxon test. Categorical data are presented as frequency with proportion and analyzed using Chi-square test or Fisher’s exact test. OS and PFS were estimated with the Kaplan-Meier method and log-rank test. *p*-Values <0.05 were considered statistically significant. Statistical analyses and PSM were conducted using SPSS version 25.0 (IBM SPSS, Inc, Chicago, IL, USA).

## Results

### Baseline

Between January 2019 and January 2021, a total of 100 patients with advanced HCC in our hospital were enrolled in the present study, of which 35 received the combined therapy and 65 patients received sorafenib monotherapy during a median follow-up of 8.8 months (range, 3.9–13.0). Thirty-four pairs were matched after PSM. Baseline patient characteristics of matched patients are summarized in **Table 1**. There was no significant difference in age, sex, HBV carrier, liver cirrhosis, ECOG performance score, Child-Pugh stage, BCLC stage, AFP, TB, ALB, AST, ALT, PLT, WBC, PT, tumor size, tumor number, macrovascular invasion, extrahepatic metastasis, or previous local regional therapy between the two groups before PSM or after PSM. Patients in the combined-therapy group received a median of five treatment cycles and those in the sorafenib-only group received a median of four treatment cycles both before and after PSM.

**Table 1:** The baseline patient characteristics.

Variables	Before PSM			After PSM		
	Combined-therapy group	Sorafenib-only group	*p*	Combined-therapy group	Sorafenib-only group	*p*
	*n* = 35	*n* = 65		*n* = 34	*n* = 34	
Age (years)	53.0 ± 10.3	53.2 ± 11.6	0.917	53.1 ± 10.4	53.3 ± 9.3	0.922
Sex (male:female)	32:3	54:11	0.398	31:3	31:3	1.000
HBV carrier	33 (94.3%)	52 (80.0%)	0.106	32 (94.1%)	33 (97.1%)	1.000
Liver cirrhosis	20 (57.1%)	41 (63.1%)	0.562	20 (58.8%)	25 (73.5%)	0.200
ECOG performance score			0.723			0.742
0	7 (20.0%)	15 (23.1%)		6 (17.6%)	5 (14.7%)	
1	28 (80.0%)	50 (76.9%)		28 (82.4%)	29 (85.3%)	
Child-Pugh stage			0.485			1.000
A	2 (5.7%)	8 (12.3%)		2 (5.9%)	3 (8.8%)	
B	33 (94.3%)	57 (87.7%)		32 (94.1%)	31 (91.2%)	
BCLC stage			0.364			0.417
B	11 (31.4%)	15 (23.1%)		11 (32.4%)	8 (23.5%)	
C	24 (68.6%)	50 (76.9%)		23 (67.6%)	26 (76.5%)	
AFP			0.950			0.462
<400 ng/ml	17 (48.6%)	32 (49.2%)		16 (47.1%)	13 (38.2%)	
≥400 ng/ml	18 (51.4%)	33 (50.8%)		18 (52.9%)	21 (61.8%)	
TBIL (μmol/L)	20.0 (12.2–29.7)	19.2 (13.5–31.1)	0.883	20.0 (12.2–29.7)	18.1 (14.1–30.7)	0.695
ALB (g/L)	38.9 ± 4.6	39.1 ± 5.4	0.809	38.7 ± 4.5	39.8 ± 4.8	0.348
AST (IU/L)	56.6 (44.8–98.2)	74.1 (47.9–105.6)	0.152	56.6 (44.8–98.2)	74.4 (48.2–112.2)	0.336
ALT (IU/L)	52.5 (40.7–65.2)	48.8 (36.4–66.9)	0.968	52.5 (40.7–65.2)	43.6 (34.9–66.0)	0.300
PLT (10^9^/L)	127.0 (81.0–181.0)	145.0 (86.0–200.0)	0.432	127.0 (81.0–181.0)	163.5 (86.8–221.0)	0.149
WBC (10^9^/L)	5.5 (4.4–8.4)	6.0 (4.3–7.5)	0.900	5.5 (4.4–8.4)	6.2 (5.0–7.6)	0.349
PT (s)	12.0 (11.4–12.8)	12.0 (11.3–12.7)	0.696	12.0 (11.4–12.8)	12.0 (11.3–12.6)	0.606
Tumor size (cm)	6.7 ± 3.6	7.5 ± 4.1	0.318	6.8 ± 3.6	8.2 ± 4.6	0.200
Tumor number			0.316			0.618
Solitary	12 (34.3%)	29 (44.6%)		12 (35.3%)	14 (41.2%)	
Multiple	23 (65.7%)	36 (55.4%)		22 (64.7%)	20 (58.8%)	
Macrovascular invasion	15 (42.9%)	33 (50.8%)	0.450	15 (44.1%)	19 (55.9%)	0.332
Extrahepatic metastasis	22 (62.9%)	40 (61.5%)	0.897	21 (61.8%)	23 (67.6%)	0.612
Previous local regional therapy						
Surgery	11 (31.4%)	11 (16.9%)	0.095	10 (29.4%)	8 (23.5%)	0.582
Ablation	1 (2.9%)	2 (3.1%)	0.950	1 (2.9%)	1 (2.9%)	1.000
TACE	18 (47.4%)	33 (50.8%)	1.000	17 (50.0%)	20 (58.8%)	0.465

PSM, Propensity Score Matching; HBV, hepatitis B virus; ECOG, Eastern Cooperative Oncology Group; BCLC, Barcelona-Clinic Liver Cancer; AFP, α-fetoprotein; TBIL, total bilirubin; ALB, albumin; AST, aspartate aminotransferase; ALT, alanine aminotransferase; PLT, platelet count; WBC, white blood cell; PT, prothrombin time; TACE, transarterial chemoembolization.

### Treatment Efficacy

**Figure 1:**
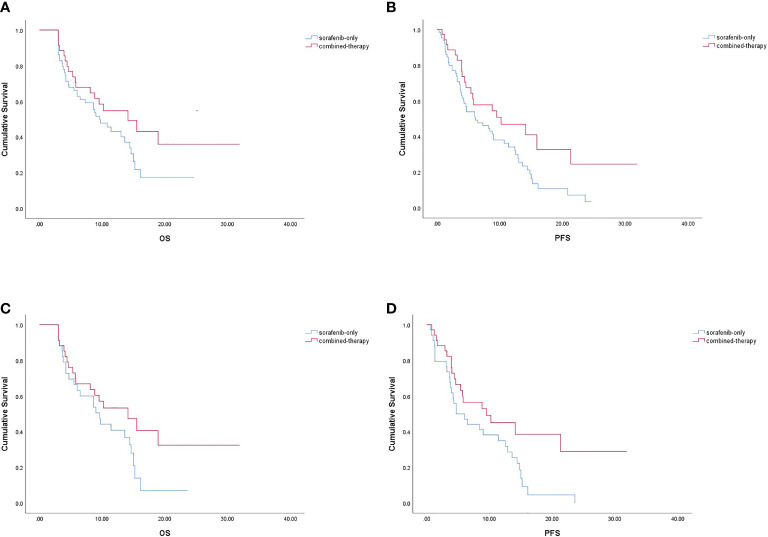
Kaplan-Meier survival curve. OS **(A)** and PFS **(B)** in the combined-therapy group and the sorafenib-only group before PSM. OS **(C)** and PFS **(D)** in the combined-therapy group and the sorafenib-only group after PSM. OS, overall survival; PFS, progression-free survival.

No CR was observed in either group (**Table 2**). Before PSM, the ORR of the combined-therapy group was significantly higher than that of the sorafenib-only group (17.1% *vs*. 3.1%, *p* = 0.037). The DCR was 68.6% in the combined-therapy group and 72.3% in the sorafenib-only group, showing no significant difference between the two groups (*p* = 0.695). There was no significant difference in OS between the two groups, with median OS of 14.1 months (6.8–21.4 months) in the combined-therapy group and 9.6 months (6.7–12.5 months) in the sorafenib-only group (*p* = 0.135). However, the combined-therapy group exhibited significantly prolonged PFS compared with the sorafenib-only group (10.2 months (95% CI: 4.5–19.0 months) *vs*. 6.1 months (95% CI: 2.5–9.7 months), *p* = 0.041) (**Figures 1A, B**).

**Table 2:** Tumor responses for patients with advanced hepatocellular carcinoma.

Response	Before PSM			After PSM		
	Combined-therapy group	Sorafenib-only group	*p*	Combined-therapy group	Sorafenib-only group	*p*
Objective response	6 (17.1%)	2 (3.1%)	0.037	6 (17.6%)	0 (0.0%)	0.010
Disease control	24 (68.6%)	47 (72.3%)	0.695	24 (70.6%)	24 (70.6%)	1.000
Complete response	0 (0.0%)	0 (0.0%)	1.000	0 (0.0%)	0 (0.0%)	1.000
Partial response	6 (17.1%)	2 (3.1%)	0.037	6 (17.6%)	0 (0.0%)	0.010
Stable disease	18 (51.4%)	45 (69.2%)	0.079	18 (52.9%)	24 (70.6%)	0.134
Progressive disease	11 (31.4%)	18 (27.7%)	0.695	10 (29.4%)	10 (29.4%)	1.000

Similar results were observed after PSM. The ORR was 17.6% for the combined-therapy group and 0.0% for the sorafenib-only group (*p* = 0.010). The DCR was the same (70.6%) for both groups (*p* = 1.000). The median OS was 14.1 months (95% CI: 7.2–21.0 months) for the combined-therapy group, which was comparable (*p* = 0.105) with that of the sorafenib-only group (median OS: 9.6 months, 95% CI: 6.1–13.1 months). The median PFS of the combined-therapy group was 9.5 months (95% CI: 1.2–17.8 months), which was significantly (*p* = 0.043) longer than that of the sorafenib-only group (4.7 months; 95% CI: 1.6–7.8 months) (**Figures 1C, D**).

### Adverse Events

All recorded treatment-related AEs are listed in **Table 3**. The most common AEs were hand and foot syndrome, thrombocytopenia, and hyperbilirubinaemia in the combined-therapy group and hand and foot syndrome in the sorafenib-only group. Before PSM, ALT was elevated in eight patients in the combined-therapy group (250.2 ± 167.6 IU/L) and six patients in the sorafenib-only group (121.2 ± 56.6 IU/L). AST was elevated in 11 patients in the combined-therapy group (310.3 ± 340.2 IU/L) and 13 patients in the sorafenib-only group (340.2 ± 352.8 IU/L). After PSM, ALT was elevated in eight patients in the combined-therapy group (250.2 ± 167.6 IU/L) and four patients in the sorafenib-only group (137.8 ± 64.4 IU/L). AST was elevated in 11 patients in the combined-therapy group (310.3 ± 182.9 IU/L) and seven patients in the sorafenib-only group (411.4 ± 439.2 IU/L). Elevated transaminase was the most frequent grade 3/4 AEs observed in both groups (before and after PSM). The combined-therapy group showed significantly higher incidence of thrombocytopenia (*p* = 0.005) and anemia (*p* = 0.040) before PSM and thrombocytopenia (*p* = 0.011) after PSM than the sorafenib-only group. However, most of these AEs were grade 1 or 2, which were easily alleviated after dose adjustment and supportive treatment. Dose modifications or treatment interruptions due to AEs were similar in the combined-therapy group and the sorafenib-only group (42.9% *vs*. 41.5%, *p* = 0.899 before PSM; 42.9% *vs*. 41.2%, *p* = 0.806 after PSM). Those with sorafenib dose reduction in both groups were comparable (28.6% *vs*. 16.9%, *p* = 0.173 before PSM; 29.4% *vs*. 14.7%, *p* = 0.144 after PSM). No treatment-associated death occurred in this study.

**Table 3:** Treatment related adverse events.

Adverse events	Before PSM						After PSM					
	Combined-therapy group		Sorafenib-only group		*p*		Combined-therapy group		Sorafenib-only group		*p*	
	Any grade	Grades 3–4	Any grade	Grades 3–4	Any grade	Grades 3–4	Any grade	Grades 3–4	Any grade	Grades 3–4	Any grade	Grades 3–4
All	33 (94.3%)	14 (40.0%)	59 (90.8%)	27 (41.5%)	0.817	0.881	32 (94.1%)	14 (41.2%)	30 (88.2%)	13 (38.2%)	0.669	1.000
Hand and foot syndrome	21 (60.0%)	2 (5.7%)	34 (52.3%)	4 (6.2%)	0.461	1.000	20 (58.8%)	2 (5.9%)	16 (47.1%)	0 (0.0%)	0.331	0.473
Hypertension	3 (8.6%)	0 (0.0%)	8 (12.3%)	2 (3.1%)	0.815	0.765	3 (8.8%)	0 (0.0%)	3 (8.8%)	1 (2.9%)	1.000	1.000
Diarrhea	10 (28.6%)	4 (11.4%)	23 (35.4%)	2 (3.1%)	0.489	0.216	9 (26.5%)	4 (11.8%)	13 (38.2%)	1 (2.9%)	0.300	0.353
Rash	8 (22.9%)	1 (2.9%)	7 (10.8%)	1 (1.5%)	0.106	1.000	7 (20.6%)	1 (2.9%)	3 (8.8%)	1 (2.9%)	0.171	1.000
Fatigue	8 (22.9%)	0 (0.0%)	18 (27.7%)	0 (0.0%)	0.599	1.000	7 (20.6%)	0 (0.0%)	7 (20.6%)	0 (0.0%)	1.000	1.000
Abdominal pain	4 (11.4%)	0 (0.0%)	12 (18.5%)	3 (4.6%)	0.360	0.499	4 (11.8%)	0 (0.0%)	5 (14.7%)	0 (0.0%)	1.000	1.000
Nausea/vomiting	3 (8.6%)	0 (0.0%)	5 (7.7%)	0 (0.0%)	1.000	1.000	3 (8.8%)	0 (0.0%)	1 (2.9%)	0 (0.0%)	0.606	1.000
Fever	2 (5.7%)	1 (2.9%)	4 (6.2%)	0 (0.0%)	1.000	0.752	2 (5.9%)	1 (2.9%)	1 (2.9%)	0 (0.0%)	1.000	1.000
Cough	2 (5.7%)	0 (0.0%)	3 (4.6%)	0 (0.0%)	1.000	1.000	2 (5.9%)	0 (0.0%)	1 (2.9%)	0 (0.0%)	1.000	1.000
Gingival hemorrhage	3 (8.6%)	0 (0.0%)	2 (3.1%)	0 (0.0%)	0.471	1.000	3 (8.8%)	0 (0.0%)	1 (2.9%)	0 (0.0%)	0.606	1.000
Elevated transaminase	11 (31.4%)	5 (14.3%)	13 (20.0%)	8 (12.3%)	0.202	1.000	11 (32.4%)	5 (14.7%)	7 (20.6%)	5 (14.7%)	0.410	1.000
Hyperbilirubinemia	14 (40.0%)	4 (11.4%)	19 (29.2%)	7 (10.8%)	0.275	1.000	14 (41.2%)	4 (11.8%)	13 (38.2%)	5 (14.7%)	0.804	1.000
Leukopenia	8 (22.9%)	1 (2.9%)	8 (12.3%)	1 (1.5%)	0.170	1.000	8 (23.5%)	1 (2.9%)	3 (8.8%)	0 (0.0%)	0.100	1.000
Thrombocytopenia	17 (48.6%)	1 (2.9%)	14 (21.5%)	2 (3.1%)	0.005	1.000	17 (50.0%)	1 (2.9%)	7 (20.6%)	1 (2.9%)	0.011	1.000
Anemia	13 (37.1%)	0 (0.0%)	12 (18.5%)	3 (4.6%)	0.040	0.105	13 (38.2%)	0 (0.0%)	6 (17.6%)	3 (8.8%)	0.059	0.238

## Discussion

The continuous emergence of new agents for systemic treatment represents a major breakthrough in the management of patients with advanced HCC. Although sorafenib as a multikinase inhibitor has been approved as the first-line systemic treatment against advanced HCC for a decade, its survival benefit is limited with a low response rate (9). Recently, the combination of molecular targeted therapy with immunotherapy has attracted tremendous interest due to their potential to improve the therapeutic efficacy compared with monotherapy (20, 21). To the best of our knowledge, this is the first study to analyze the efficacy and safety of camrelizumab in combination with sorafenib in patients with advanced HCC. Our results demonstrated that the combined therapy showed superiority over the sorafenib monotherapy in terms of PFS and ORR, although it did not show an OS benefit.

Different combined treatment modalities for advanced HCC have been discussed due to limited clinical benefits of monotherapy (22). The recent introduction of immunotherapy has demonstrated promising efficacy in solid tumor treatment and various clinical trials involving immunotherapy are currently ongoing to explore its potential survival benefit in HCC patients (23, 24). However, the optimal combined regimens remain undefined despite remarkable progress has been made in systemic therapy for patients with advanced HCC. So far, there is little knowledge about the potential synergic effect between camrelizumab and sorafenib on advanced HCC.

In the present study, treatment efficacy of camrelizumab plus sorafenib in the 34 patients with advanced HCC was assessed. Results showed an ORR of 17.6%, a DCR of 70.6%, a median PFS of 9.5 months, and a median OS of 14.1 months. Clinical trials involving combination therapies of immune checkpoint inhibitors and molecular targeted therapy agents in HCC are limited. In a phase II trial, camrelizumab combined with apatinib as first-line and the second-line treatments for advanced HCC showed median PFS of 5.7 and 5.5 months and ORR of 34.3% and 22.5%, respectively (25). In a phase Ib study, lenvatinib plus pembrolizumab showed a median PFS of 8.6 months, a median OS of 22 months, and an ORR of 46% in patients with unresectable HCC (26). In a phase III IMbrave150 study (18), patients with unresectable HCC in the atezolizumab-bevacizumab group showed better clinical outcomes than those in the sorafenib group, with a median PFS of 6.8 months and an ORR of 89%. Possible reasons for such discrepancies might be related to the retrospective observational design, differences in patient baseline characteristics, and different treatment regimens. In our study, 44.1% of patients showed a macrovascular invasion, 61.8% had extrahepatic metastasis, and 52.9% had a baseline AFP >400 ng/ml, while only 29.4% had received a surgery. Lacking effective postprogression therapy could be another explanation for the relatively short OS in our study. Although limitations should be considered when interpreting results of this study, our findings provided insight into potential therapeutic strategies for advanced HCC.

Despite the great promise of molecular targeted therapy agents, their clinical benefits are limited due to tumoral heterogeneity and acquired resistance (27, 28), emphasizing the necessity of exploring combination therapies to improve the therapeutic efficacy. Our findings showed that the addition of camrelizumab to sorafenib was associated with prolonged PFS and higher ORR, indicating that the combination of immunotherapy and targeted therapy could enhance antitumor benefit. Preclinical studies have demonstrated that antiangiogenic agents targeting VEGF/VEGFR could inhibit tumor growth and metastasis (28, 29). In addition, angiogenesis inhibitors possess immunomodulatory effects, including increasing T-cell activity and promoting T-cell infiltration (30). On the other hand, vasculature normalization *via* inhibition of angiogenesis could reduce tumor hypoxia, improve drug delivery, and facilitate immune cell infiltration (31). Therefore, targeted therapy could reprogram an immunosuppressive tumor microenvironment into an immunostimulatory environment, thereby contributing to enhanced antitumor immunity (17). More studies are needed to investigate the underlying mechanism involved in the enhanced antitumor effect of a combination therapy and identify patients who will benefit the most from such combination.

The most common sorafenib-related AEs were in accordance with those observed in previous reports. Here, any-grade AEs of thrombocytopenia occurred more frequently in the combined-therapy group compared with the sorafenib-only group, which might be related to the utilization of camrelizumab. Although the combination therapy showed increased hematologic toxicities, the incidence of grade 3/4 toxicities was comparable between the two treatment groups. Moreover, most AEs in both groups were mild to moderate in severity, and no significant difference in the incidence of dose adjustments or treatment interruptions was observed between the two groups. The current study showed that side effects of the combined therapy were generally controllable and tolerable.

Several limitations in this study need to be addressed. First, the retrospective design of a retrospective study might have introduced potential biases, although PSM was conducted to reduce potential selection bias. Second, this study was based on a single-center experience with a relatively small number of patients. Third, the follow-up period was relatively short. Furthermore, the heterogeneous individual therapeutic response highlights the need to understand who will respond better to the treatment.

## Conclusion

In conclusion, camrelizumab combined with sorafenib appears to be a promising therapeutic strategy in the management of advanced HCC, which showed prolonged PFS, higher ORR with well-tolerated AEs compared with sorafenib monotherapy. These results offer our preliminary experience in combination strategies for advanced HCC, which are informative for clinical decision making. Nevertheless, further prospective randomized controlled studies with larger sample sizes and longer follow-up time are warranted to support these preliminary findings of the study.

## Data Availability Statement

The original contributions presented in the study are included in the article/supplementary material, further inquiries can be directed to the corresponding author/s.

## Ethics Statement

Written informed consent was obtained from the individual(s) for the publication of any potentially identifiable images or data included in this article.

## Author Contributions

QL, NY, JL, and LZ contributed to the study conception and design. QL, NY, JL, KW, and XP collected the data. QL analyzed the data. QL drafted the manuscript, and the other authors revised the manuscript. All authors contributed to the article and approved the submitted version.

## Funding

This study was supported by the Medical Research Project jointly funded by Chongqing Science and Technology Commission and Chongqing Health Commission (2019ZDXM046), the Technological Innovation and Application Demonstration Special Project of Chongqing (cstc2018jscx-mszdX0012), and the Scientific and Technological Innovation Special Project of Army Medical University (2019XLC2006).

## Conflict of Interest

The authors declare that the research was conducted in the absence of any commercial or financial relationships that could be construed as a potential conflict of interest.

## Publisher’s Note

All claims expressed in this article are solely those of the authors and do not necessarily represent those of their affiliated organizations, or those of the publisher, the editors and the reviewers. Any product that may be evaluated in this article, or claim that may be made by its manufacturer, is not guaranteed or endorsed by the publisher.

## Abbreviations

PSM, propensity score matching; PFS, progression-free survival; OS, overall survival; AEs, adverse effects; ORR, overall response rate; DCR, disease control rate; HCC, Hepatocellular carcinoma; TKIs, Multitarget tyrosine kinase inhibitors; VEGFRs, vascular endothelial growth factor receptors; PDGFRs, platelet-derived growth factor receptors; FGFRs, fibroblast growth factor receptors; c-KIT, stem cell factor receptor; RET, glial cell-derived neurotrophic factor receptor; OS, overall survival; ICIs, Immune checkpoint inhibitors; CTLA-4, cytotoxic T-lymphocyte antigen-4; PD-1, programmed cell death protein1; PD-L1, programmed cell death protein ligand; FDA, Food and Drug Administration; AASLD, American Association for the Study of Liver Diseases; ECOG, Eastern Cooperative Oncology Group; mRECIST, modified Response Evaluation Criteria in Solid Tumors; HBV, hepatitis B virus; BCLC, Barcelona Clinic Liver Cancer; AFP, alpha-fetoprotein; TBIL, total bilirubin; ALB, albumin; AST, aspartate aminotransferase; ALT, alanine aminotransferase; PLT, platelet count; WBC, white blood cell; PT, prothrombin time; CR, complete response; PR, partial response; SD, stable disease; PD, progressive disease; ORR, objective response rate; DCR, disease control rate; CTCAE, Common Terminology Criteria for Adverse Events.
